# IP6: From Seeds to Science—A Natural Compound’s Path to Clinical Promise

**DOI:** 10.3390/biom15121652

**Published:** 2025-11-26

**Authors:** Alissa Saverino, AbulKalam M. Shamsuddin, Ivana Vucenik

**Affiliations:** 1 Department of Epidemiology and Human Genetics, University of Maryland School of Medicine, Baltimore, MD 21201, USA; alissa.saverino@som.umaryland.edu; 2 IP-6 Research, Inc., Lutherville, MD 21093, USA; research@ip-6.net; 3 Department of Medical and Research Technology, University of Maryland School of Medicine, Baltimore, MD 21201, USA; 4 Department of Pathology, University of Maryland School of Medicine, Baltimore, MD 21201, USA

**Keywords:** inositol, phytic acid, cancer, mechanism

## Abstract

Inositol hexaphosphate (IP6), also known as phytic acid, has historically been classified as an antinutrient due to its mineral-chelating properties, which were believed to impair nutrient absorption. Early reports fueled concerns that high dietary phytate intake could contribute to mineral deficiencies, albeit without direct scientific evidence, particularly in populations lacking dietary diversity. However, lifetime animal experiments have demonstrated that IP6 does not have any negative effect on mineral bioavailability and if there is any in humans, it is largely context-dependent. Even more importantly, beyond nutritional implications, IP6 has emerged as a bioactive molecule with promising therapeutic potential across various cancer types and clinical conditions. Preclinical and clinical research indicate that IP6, alone or in tandem with inositol (Ins), selectively targets cancer cells and enhances chemotherapy efficacy. Growing evidence also suggests that IP6 plays a protective role in cardiovascular health, neurodegenerative disorders, and metabolic diseases. While clinical trials remain limited, extensive in vitro, in vivo, and epidemiological studies support a shift in how IP6 is perceived among the scientific community—moving from an antinutrient to a health-promoting compound. As research progresses, further clinical investigations are essential to fully elucidate IP6’s therapeutic applications and its benefit to disease prevention.

## 1. Introduction

Inositol hexaphosphate (IP6), also referred to as phytic acid or phytate, is a naturally occurring compound predominantly found in seeds, cereals, legumes, and nuts. Nearly all mammalian cells also contain high concentrations of IP6, where it is a key contributor in processes such as signal transduction, regulation of cell proliferation and differentiation, DNA repair, maintaining cellular homeostasis and more [1]. Its biochemical structure, first depicted by Anderson (1914) [2], consists of an inositol ring fully phosphorylated with six phosphate groups. This configuration grants IP6 distinct features, including antioxidant capabilities and significant capacity for chelating essential minerals such as calcium, iron, and zinc [3,4]. This chelation ability has historically positioned IP6 in a controversial light, persistently labeled by some as an antinutrient. This term has long overshadowed its broader potential in promoting human health, despite growing evidence of its promising clinical implications.

The notion that IP6 may induce malnutrition stems from its convoluted relationship with nutrient absorption. Within the gastrointestinal tract, IP6 has demonstrated the ability to bind to trace elements and minerals, which may hinder their bioavailability [5]. These binding properties have raised concerns regarding IP6’s potential to augment nutrient deficiencies in certain at-risk populations, such as those with limited dietary diversity [6]. For instance, malnourished individuals often face impaired absorption due to gastrointestinal inflammation—this insufficient nutrient absorption can therefore be magnified by IP6’s presence for these at-risk groups [7]. Conversely, others have evidenced that, when consumed amid a well-balanced diet, IP6 has a negligible effect on mineral bioavailability [6,8,9]. Modern observations have revealed that this antinutrient status of IP6 is in fact context-dependent, perhaps fueling a prospective shift in scientific perspective around its capacity to support human wellness. This review aims to provide a comprehensive overview of IP6, tracing its evolution from a misunderstood antinutrient to a multifunctional bioactive compound, as well as to elucidate its molecular mechanisms, therapeutic promise, and implications for future clinical research.

## 2. The Antinutrient Debate

The enduring antinutrient debate involving IP6 largely emerged from early studies that noted its mineral-binding qualities could be detrimental to nutrient absorption. IP6 chelates minerals such as calcium, iron, and zinc, resulting in the formation of insoluble complexes and subsequently impeding on mineral absorption during digestion [5]. Investigations from the mid-20th century, such as those by McCance and Widdowson (1942), observed that diets rich in phytate notably reduced mineral bioavailability [10,11]. Both studies strategically controlled dietary conditions and assessed nutrient absorption across 6–8 participants, examining the impact of diets based on white (low in phytate) and brown (high in phytate) breads, alongside dephytinized variants. Those consuming brown breads exhibited hindered nutrient absorption, particularly of calcium [11], while those who ate dephytinized bread demonstrated enhanced absorption of calcium and magnesium [10].

Further research underscored the variability in IP6’s impact, with factors such as dietary calcium levels influencing the extent of its mineral-binding qualities [12]. Cruickshank et al. (1945) found a range of phytate hydrolysis among individuals, with some displaying near-complete breakdown of IP6 during digestion [13]. These early findings sparked worry, particularly for populations heavily reliant on cereals and legumes, where limited dietary diversity elevated the risk of nutrient deficiencies [14]. Consequently, the term ‘antinutrient’ became widely associated with IP6 (“guilt by association”) without direct scientific evidence, fostering a negative perception that dominated scientific and public discourse for decades. This skepticism prompted substantial adjustments in food processing to minimize IP6 content and thereby mitigate its effects on mineral bioavailability. While these interventions addressed certain nutritional concerns, they also reinforced the broader narrative of IP6 as a dietary inhibitor.

However, modern research has challenged this view, discovering potent health benefits of IP6 that extend well beyond its role in mineral metabolism. It has been established that the antinutritional aspects of IP6 are not universal, as mineral concentration and overall dietary composition modulate its mineral-binding activity [15]. Additionally, food components including organic acids, vitamin C, and fermented products, have been shown to effectively counteract IP6-inhibited absorption through competitively binding to trace elements and minerals [16,17]. This knowledge, coupled with the lack of evidence from well-nourished populations that suggests IP6 intake may undermine nutrient absorption, urges the scientific community to reevaluate the long-standing antinutrient label assigned to IP6.

## 3. Modern Discoveries—Shifting Perspectives

**Figure 1:**
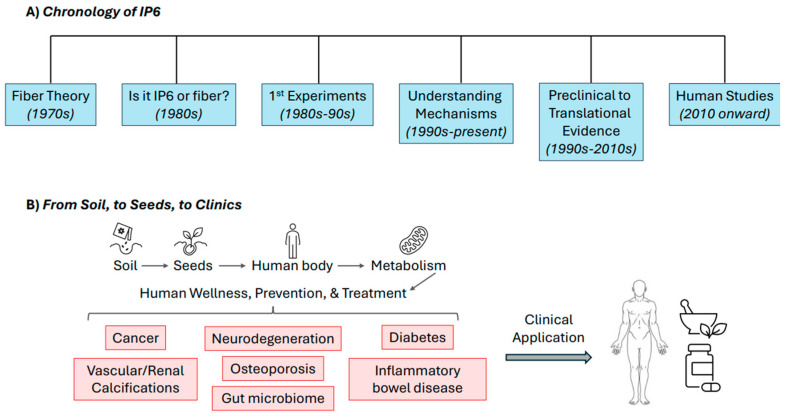
Historical and translational trajectory of IP6 research. (**A**) Chronology of IP6. Key milestones in the development of IP6 research, beginning with the fiber theory in the 1970s, followed by debates over whether its effects were due to fiber or IP6 (1980s), first experimental studies in mice and rats (1980s–1990s), mechanistic discoveries (1990s–present), preclinical to translational evidence across multiple cancer models (1990s–2010s), and the initiation of human studies (2010 onward). (**B**) From soil, to seeds, to clinics. Conceptual framework illustrating the path from soil, to seeds, to human metabolism, and ultimately, to clinical application. IP6 contributes to wellness, prevention, and treatment across diverse health conditions, including cancer, neurodegeneration, diabetes, vascular and renal calcifications, osteoporosis, inflammatory bowel disease, and modulation of the gut microbiome.

Recent decades have embodied a shift in the field’s approach to IP6 research, with many investigations focused on its health-promoting potential. This evolution is illustrated in Figure 1, outlining the chronological milestones and translational pathway of IP6 research from seeds, to soil, to clinical relevance. Central to this transformation were the initial preclinical studies conducted by Shamsuddin et al. in the 1980s and 1990s [18,19,20], followed by mammary tumor inhibition using different animal models and carcinogens [21,22]—not only did these investigations showcase IP6’s anticancer functions, but also confirmed that life-time consumption of IP6±Inositol did not cause any reduction in Ca, Mg, Fe, or Zn levels in the blood or bones of rats and mice [21]. The earliest work involved rat and mouse colon cancer models with IP6 administered via drinking water, where it prevented the malignancy in both models in a dose-dependent manner [18,19]. These pioneering experiments were based upon the ‘fiber theory’, which suggested that high-fiber diets could protect against colon cancer, and were conducted in response to epidemiological data showing that diets rich in IP6-containing foods, such as cereals and legumes, were inversely associated with the disease [18,19]. Shamsuddin postulated that orally administered IP6 could be absorbed through the gastrointestinal tract and dephosphorylated by phytases and phosphatases into lower inositol phosphates (IPs) which would then enter the intracellular IP pool and thereby influence tumor initiation and advancement [18,19]. This was a novel concept, as it was widely believed that the highly negatively charged IP6 molecule was incapable of crossing the intestinal barrier. He furthermore hypothesized that combining IP6 with inositol (Ins) could strengthen IP6’s anticancer properties by prompting the generation of a greater amount of lower IPs. Given the fact that these IPs operate as key signaling molecules in nearly all mammalian cells, it was anticipated that the resulting anticancer effects would transcend across multiple cell types and tissues.

Shamsuddin et al. showed that IP6 mitigated the amount and size of large intestinal tumors, even when administered 5 months after the carcinogen exposure [19]. Specifically, animals treated with IP6 had 27% lesser tumors as compared to those untreated [19]. This discovery marks a pivotal moment in IP6 research, as it catalyzed a surge of interest in IP6’s biological mechanisms and how they may be harnessed for cancer treatment. Subsequent studies in the 1990s supported these findings, where Drs. Pretlow and Reddy unveiled that IP6 treatment lessened the incidence of aberrant crypts, a hallmark biomarker for colon cancer at the time [23,24]. It was then documented that IP6 increases the expression of p53, a major tumor suppressor, in human colon carcinoma cells [25]. Concordantly, IP6 substantially reduced α-fetoprotein (AFP) release in human hepatoma cells, reinforcing that IP6 acted on tumor-associated markers beyond tumor suppressors such as p53, but also secreted oncofetal proteins across diverse models [26].

Amid the 1990s and early 2000s, research on IP6 expanded into other cancer types, including prostate, liver, and pancreatic. These investigations propelled the field’s knowledge of IP6 and its protective mechanisms. Resultingly, a large body of in vitro and in vivo work firmly established IP6 as a broad-spectrum antineoplastic agent across a vast array of cell and tissue systems. Throughout these models, IP6 persistently produced a clear time- and dose-dependent growth-inhibitory response at concentrations ranging from 0.5 to 5.0 mM [26,27,28,29,30,31].

Uncontrolled, self-sustaining cellular proliferation epitomizes a fundamental feature of cancer biology. Potent antiproliferative effects of extracellular and exogenous IP6 have been reported in human leukemia [27,28], colon [29], cervical [32], prostate [30,31], and hepatoma cells [26]. Aside from these findings in epithelial cells, such antiproliferative observations also extend to mesenchymal tumors, including fibrosarcoma (murine) and rhabdomyosarcoma (human) [33,34]. Notably, leukemia and hepatocellular carcinoma cells exhibit a pronounced sensitivity to IP6, suggesting model-specific mechanisms underlying the growth-inhibitory responses [35].

These early findings laid the groundwork for deeper investigation into the mechanisms driving IP6’s anticancer functions. Treatment of human breast cancer cell lines with IP6 indicated modulation of several disease-related molecules and signaling pathways—IP6 inhibited proliferative pathways, activated pro-apoptotic and anti-proliferative pathways, induced G0/G1 cell cycle arrest, and reduced retinoblastoma protein phosphorylation [3,36]. IP6 has also been shown to promote differentiation and maturation of malignant cells, frequently accompanied by partial reversion toward a more normal phenotype, as evidenced in K-562 hematopoietic cells [27], HT-29 colon cancer cells [29], prostate [30], and rhabdomyosarcoma models [34]. As the ensuing years gave rise to similar compelling discoveries, a number of comprehensive reviews have since been published outlining IP6’s range of anticancer functions [3,4,37].

**Figure 2:**
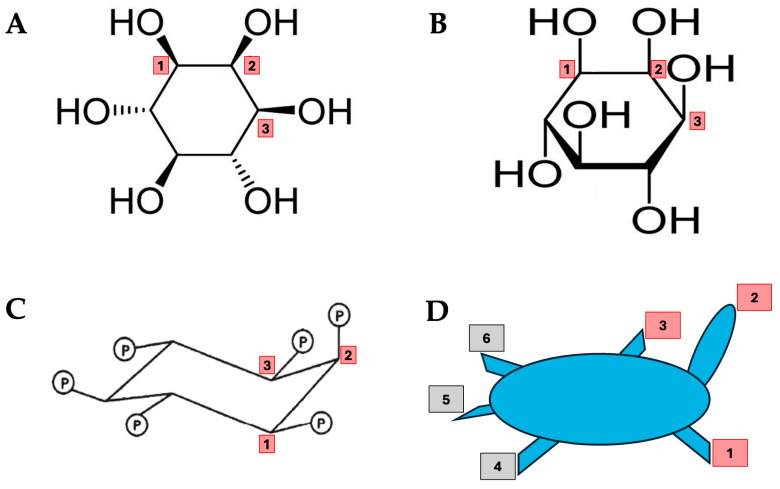
Structures of IP and myo-inositol. (**A**) myo-Inositol is represented as a wedge-dash notation. (**B**) Haworth projection; (**C**) Chair conformation of myo-inositol hexaphosphate (IP phytic acid) with the unique configuration of phosphate groups in positions 1, 2 and 3 (axial–equatorial–axial) and (**D**) schematically as a turtle.

At this time, IP6 had already been recognized as a natural antioxidant, as publications such as Graf and Eaton (1990) demonstrated its capacity to neutralize free radicals and protect cells from oxidative damage [38]. The inherent chelation ability of IP6, originally cited as the basis for concerns about nutrient depletion, is key to its powerful antioxidant properties [39]. IP6 is structurally characterized by phosphate groups positioned uniquely at the 1, 2, and 3 axial–equatorial–axial configuration, a feature exclusive to this compound (Figure 2). This molecular arrangement enables a high-affinity interaction with iron, effectively blocking its catalytic role in hydroxyl radical formation and thereby fortifying IP6’s antioxidant capacity [38]. IP6 and its lower phosphorylated derivatives are also present, though in smaller quantities, in nearly all mammalian cells, where they play central roles in cellular physiology. These inositol phosphates participate in fundamental processes such as cell cycle regulation, differentiation, ion channel function, and embryonic development, and are particularly vital as modulators of intracellular signaling pathways [40,41,42,43].

This new wave of scientific inquiry continued to uncover the cellular activity of IP6 that drives its health-promoting benefits. Mechanistic investigations progressed throughout the years, showcasing the underlying mechanisms of IP6’s anticancer effects, many of which being rooted in cellular signaling. A constellation of molecular pathways and intracellular targets have since been implicated in relation to this compound. For instance, IP6 was shown to interfere with receptor interactions by sterically blocking the heparin-binding domain of basic fibroblast growth factor, thereby disrupting downstream signaling cascades [44]. Its impact spans major cellular signaling pathways, including inhibition of phosphatidylinositol-3 kinase (PI3K), modulation of protein kinase C (PKC) and mitogen-activated protein kinases (MAPKs) [36,45,46,47].

These upstream effects translate into diverse functional outcomes: IP6 downregulates cell cycle regulators such as p21 and p27, inhibits phosphorylation of retinoblastoma protein (pRB), and suppresses PI3K/protein kinase B (PI3K/Akt) and protein kinase C/RAS/extracellular signal-regulated kinase (PKC/RAS/ERK) signaling pathways [36,37,48]. Such modulation is accompanied by decreased NF-kB activity and inflammatory responses, further implicating IP6 as a multifunctional agent within the tumor microenvironment [37,48]. Additionally, IP6 has been shown to inhibit angiogenesis via downregulation of vascular endothelial growth factor (VEGF) expression, both on the protein and mRNA level, in HepG2 liver cancer cells [49]. Other downstream effects include reduced metastatic potential and altered expression of key cellular genes such as p53, BCL-2, and MMPs. More recently, IP6 has been implicated in regulating Cullin-RING E3 ligase dynamics, suggesting an even broader scope of intracellular control [50].

Within the last decade, IP6 research has flourished, offering greater clarity into its pharmacological properties and multifaceted role in preventing and managing a wide spectrum of chronic conditions. IP6 has demonstrated protective effects against cardiovascular, neurodegenerative, and inflammatory bowel diseases [51,52]. The related mechanisms through which IP6 combats these varying conditions are linked to its ability to regulate inflammation, modulate immune responses, and influence the gut microbiome. IP6 alters macrophage polarization, shifting them from a pro- to anti-inflammatory state; these macrophages also presented with gene expression signatures linked to an anti-inflammatory response [53]. Furthermore, IP6 reduces the secretion of pro-inflammatory cytokines (e.g., IL-8) via impeding on MAP kinase activity [54]. The profound immunoregulatory effects of IP6 have been repeatedly documented, wherein it enhances natural killer (NK) cell activity, correlating with suppressed tumor development in colon cancer models [55,56]. Aligning with the field’s exponential intrigue with the gut microbiome, researchers have implemented robust technologies to study how IP6 might influence these microbes. IP6-treated colorectal cancer mouse models exhibit a restoration of favorable gut bacteria, and a decrease in pathogenic counterparts associated with increased inflammation and disease progression [57]. Transcriptomic analysis also revealed differential gene expression in IP6-treated mice linked to minimized liver metastasis [57]. With mounting evidence that IP6 supports health across a diverse range of chronic ailments, its role appears to transcend disease prevention, perhaps underscoring its importance in sustaining core physiological functions imperative for human well-being.

## 4. IP6 as a Therapeutic Agent—Clinical Insights and Emerging Potential

The therapeutic potential of IP6 was initially recognized in a transplantable and metastatic murine fibrosarcoma model (FSA-1), marking the earliest evidence that IP6 could operate as an effective anticancer agent able to control tumor metastasis and spreading. In this model, intraperitoneal injections of IP6 led to substantial decrease in primary tumor size and improved overall survival [33]. This model also revealed that IP6 could significantly suppress the development of pulmonary metastases when administered following an intravenous inoculation of FSA-1 cells [33]. These observations yielded early confirmation that IP6 could dually impact tumor initiation, along with progression and metastatic spread. Aiming to build upon these novel insights, two additional models were developed to further interrogate IP6’s efficacy in mitigating tumor formation and promoting regression of established tumors.

**Figure 3:**
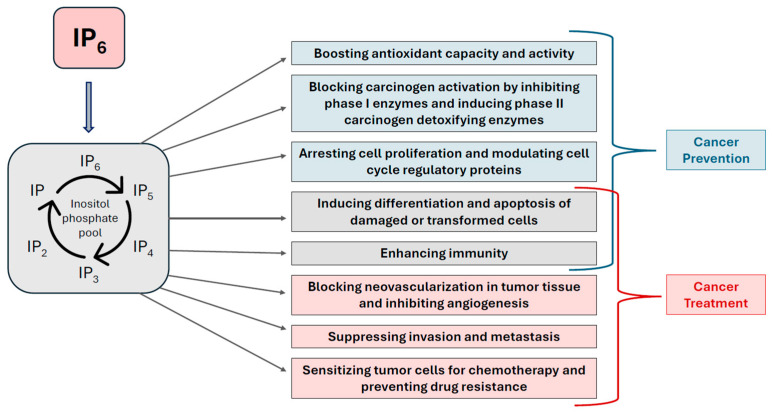
Biochemical mechanistic basis of IP6 in cancer prevention and treatment. IP6 enters the inositol phosphate pool, subsequent to rapid intake and dephosphorylation. It is metabolized into lower phosphorylated forms (IP1–IP5) that exert diverse biological effects with roles in cancer prevention, treatment, and dual-action roles. Preventative mechanisms (blue) include boosting antioxidant capacity, inhibiting carcinogen activation, regulating cell cycle proteins. Treatment-related mechanisms (red) involve blocking angiogenesis, suppressing invasion and metastasis, and sensitizing tumor cells to chemotherapy while overcoming drug resistance. Induction of apoptosis and differentiation of damaged cells, and enhancing immunity, reflect mechanisms dually rooted in cancer prevention and treatment.

First, human rhabdomyosarcoma RD cells were subcutaneously implanted into nude mice to evaluate IP6’s ability to hinder early tumor formation. Peritumoral IP6 treatment (40 mg/kg), initiated two days after cell implantation and administered three times weekly for five weeks, resulted in a striking 49-fold reduction in tumor size [34]. The second was a liver cancer model, designed to clarify IP6’s performance in inhibiting experimental hepatoma [26,58]. When HepG2 cells were pre-treated with IP6 in vitro and later inoculated into nude mice, they failed to form tumors, indicating a complete loss of tumorigenicity resulting from a single exposure of IP6 [58]. In a parallel arm assessing tumor regression, IP6 was administered via direct intratumoral injection to preexisting liver tumors once they reached approximately 1 cm in diameter [58]. After 12 days of treatment, IP6-treated mice exhibited a 3.4-fold lesser tumor weight as compared to controls [58]. Evidence of IP6’s antitumor properties was further reinforced in prostate cancer models, where its delivery via drinking water markedly curtailed tumor growth in human xenographs [59]. Collectively, these preclinical discoveries laid a strong foundation for therapeutic investigation of IP6, illustrating its capacity to impede tumor formation, suppress growth of established cancers, and limit metastatic growth across numerous experimental systems [33,34,58,59]. Figure 3 presents a range of cellular and molecular activities relevant in cancer prevention and treatment involving IP6.

Virtually all anticancer agents pose severe side effects on healthy cells, tissues, and organs. Conversely, IP6 demonstrates a unique combination of therapeutic sensitivity and biological selectivity—potently targeting diseased cells while safeguarding normal cellular function. Marking one of the earliest investigations into IP6’s selective nature, researchers treated bone marrow and isolated CD34+ progenitor cells with IP6 and monitored the effects [28]. IP6 showed supreme efficacy against leukemic progenitors derived from chronic myelogenous leukemia patients, however it had no cytotoxic or cytostatic effects on normal bone marrow cells under the same experimental conditions [28]. In vitro comparisons of human breast cancer cell lines yielded similar results regarding IP6’s selectivity. Malignant MCF-7 and MDA-MB-231 (i.e., triple-negative breast cancer-TNBC) breast cancer cells showed significantly reduced proliferation following IP6 treatment due to increased rates of apoptosis [3]. The growth of non-cancerous MCF-10A epithelial breast cells remained unaffected, further illustrating IP6’s capacity to differentiate between cancerous and normal cells [3].

Such precision emphasizes IP6’s potential as a highly desirable therapeutic agent, as the ability to selectively target diseased cells while preserving healthy counterparts marks a chiefly sought-after quality when designing a clinically administered treatment. Notably, in Parkinson’s disease models, IP6 demonstrates an opposing effect by reducing apoptosis in neurons, offering neuroprotective benefits that mitigate the advancement of neurodegenerative disorders [60,61]. These contrasting outcomes accentuate IP6’s uniquely adaptive nature: while it induces apoptotic mechanisms in cancer cells, it simultaneously preserves and protects vulnerable healthy neurons in neurodegenerative settings. This context-specific selectivity highlights the therapeutic versatility of IP6 across distinct disease models, showing that IP6 can be adapted to almost all tailored treatment.

IP6 has also exhibited the ability to enhance the effects of anti-cancer therapies when administered in tandem. This strategy is particularly valuable in oncology, where combination therapy is often used to improve efficacy and minimize toxicity. Studies have revealed that IP6 boosts the efficacy of both doxorubicin and tamoxifen in breast cancer, demonstrating prominent potency against ER- and doxorubicin-resistant cell lines, thereby reducing the necessary drug dosage [62]. These findings carry key implications, as the ability to act synergistically with standard therapies is especially important in the context of chemoprevention and overcoming acquired drug resistance. For instance, tamoxifen is often administered as a preventive treatment in the post-therapy setting, and doxorubicin is limited by both its cardiotoxicity and the development of drug resistance. These challenges are difficult to manage clinically, but are mitigated when IP6 is combined with these agents [62]. IP6 has also been shown to reverse oxaliplatin resistance in colorectal cancer cells by counteracting ER stress-induced senescence via inhibition of PERK activation and eFF2 dipthamide modification [63].

Inositol (Ins), the precursor molecule for IP6, is largely accepted in the field of nutrition and is recognized for its health benefits, namely regarding metabolic and reproductive health [64,65]. Both IP6 and Ins have been designated as Generally Recognized As Safe (GRAS) by the U.S. Food and Drug Administration (FDA). Within the body, Ins is converted into IP6 and other inositol phosphates through a series of phosphorylation reactions predominately carried out by inositol phosphate kinases. Previous investigations established that IP6’s anticancer properties are magnified when combined with Ins [3]. Thus, the first observational clinical study was performed in colon cancer patients undergoing chemotherapy who were supplemented IP6 plus Ins [66]. The authors found that this supplementation prevented significant drops in blood cell counts, a common side effect of chemotherapy, potentially enabling uninterrupted treatment [66]. A pilot study in breast cancer patients also found that IP6 combined with Ins improved quality of life and reduced chemotherapy-related side effects [67]. In regards to Ins and its independent effects on chemoresistance, recent preclinical work has demonstrated that Ins can resensitize paclitaxel-resistant triple-negative breast cancer (TNBC) cells both in vitro and in vivo settings. Mechanistically, Ins suppressed mitochondrial fission by inhibiting AMPK activation, thus preserving mitochondrial integrity [68]. As elevated mitochondrial fission is a known feature of drug-resistant cancer cells, heightening potential for invasion and metastatic spread, this observed inhibition by Ins is quite pertinent. In xenograft mouse models, tumors in the paclitaxel plus Ins group were significantly smaller than those treated with paclitaxel alone within 21 days of treatment, and histological assays confirmed reduced tumor cell density and proliferation [68]. Importantly, no adverse effects have been reported in studies administering IP6 or Ins to humans or animal models, even at high doses [4]. That is, across both preclinical and the few clinical investigations, neither compound has demonstrated toxicity or clinically significant side effects, and their safety profile remains favorable even under prolonged or high-dose administration.

Aside from these clinical insights, anecdotal evidence from individual cases supports the potential for therapeutic employment of IP6. One of the most recent and fascinating case reports involves a patient with stage IV metastatic melanoma who chose to forgo conventional therapy and instead opted for the over-the-counter supplemental form of IP6 plus Ins. This individual reached total remission and remained in remission when evaluated 3 years later [69]. While studies have repeatedly documented the therapeutic nature of IP6, the breadth of clinical research investigating its applications remains limited. To clearly summarize the current landscape of these clinical trials, to our knowledge, there are no ongoing or prospectively planned trials evaluating IP6 ± Ins regarding their anti-cancer therapeutic utility, thereby highlighting the present gap of translational efforts despite the promising evidence observed to date.

## 5. Summarized Molecular Mechanisms of IP6

Molecular studies of the anticancer action of IP6 have been extensively studied, and we present a summary. After cellular uptake, IP6 is dephosphorylated to lower inositol phosphates and inositol, affecting activity of various regulatory cellular proteins. Additionally, through direct binding, IP6 can impact the function of different cellular enzymes. IP6 can modulate many cancer-related pathways, such as PI3K and Akt, PDH and AMPK, which are able to activate the survival pathways, upregulate aerobic glycolytic enzymes and trigger the epithelial–mesenchymal transition. IP6 was shown to inhibit cell adhesion, migration and invasion, all processes involved in cancer metastasis. Additionally, IP6 induces cell apoptosis through regulation of many pathways, including AKT/mTOR. Multiple studies have shown that IP6 is able to modulate biological processes implicated in development of various cancers, such as inflammation, apoptosis, angiogenesis, proliferation, cell signaling, and gene expression. Additionally, its ability to boost immunity and NK cells, and its antioxidant activity also contributes to its anticancer effect. Even the regulation of microRNAs in colon cancer was shown for IP6 [70]. There are numerous excellent reviews addressing the molecular aspects of anticancer effects of IP6 [4,37,52].

## 6. Redefining the Role of IP6 in Health and Nutrition—Future Directions

Early studies characterized IP6 primarily by its mineral-chelating abilities, raising concerns about its potential to hinder nutrient absorption and prompt malnutrition without scientific evidence; it was rather a “guilt by association”. Modern research, however, has revealed that these antinutrient effects are profoundly context-dependent and insignificant in the presence of a balanced diet. Furthermore, the various lifetime experiments in different animal models have shown no mineral deficiencies, nor have any published scientific paper to date reported any deficiency. On the contrary, the diverse therapeutic utility of IP6 across various chronic conditions and cancers have been well documented. These findings decisively oppose the outdated view of IP6 being a compound to avoid, ultimately redefining this molecule as a vital contributor in health promotion and disease prevention.

Recent comprehensive reviews emphasize this paradigm shift, demonstrating the integral role of dietary phytates in supporting overall wellness and reducing risk of various ailments. Pujol et al. compiled data from over 50 years of phytate research and concluded that diets such as the Mediterranean, where IP6 is one of the major components, are associated with the prevention of vascular calcifications, kidney stones, bone mass loss, cancer, neurodegeneration, and diabetes complications [71]. The authors noted there are no pharmacological treatments that directly mitigate neurological decline, vascular calcification, and urolithiasis, yet IP6 has exhibited effectiveness in addressing these conditions in in vitro, animal model, and epidemiological settings [71]. Plant-based food diets are often recommended to individuals with chronic kidney disease, where the protective effects of the diet are ascribed to IP6 [72]. The application of high-phytate diets extends beyond these conditions. Evidence also suggests these diets may offer detoxifying benefits in the context of heavy metal poisoning, as it has been utilized as a successful therapeutic modality to mitigate acute lead toxicity [73]. These phytate-rich dietary studies suggest that IP6’s benefits are not only prominent when taken as a supplement, but also when consumed via natural food sources.

A review publication from Zyla et al. delved thoroughly into this health-promoting notion in cereals high in dietary phytate [74]. The authors communicate that the consumption of phytate-rich cereals can contribute to the prevention of a variety of health conditions, from metabolic concerns to colon cancers. They emphasize phytate’s intricate role in nutrient absorption and bioactivity, where its synergistic interactions with phenolic compounds and enzymes, can contribute to enhanced metabolic and gut health [74]. Pragya et al. (2025) detailed similar metabolic advantages of phytic acid, particularly in relation to glucose regulation and anti-diabetic properties [73]. Phytic acid has been shown to modulate insulin secretion and slow starch digestion by interacting with gastric enzymes and proteins associated with carbohydrate metabolism [73]. Its ability to bind with calcium, a vital cofactor for the starch-digesting enzyme α-amylase, may lead to reduced enzymatic activity during digestion. With increasing phytic acid, the efficiency of starch hydrolysis reduces, ultimately reducing blood glucose levels [73].

Advances in the field of agricultural biotechnology have unveiled promising strategies to augment these benefits through biofortification of IP6-containing crops. Murgia et al. (2012) underscore the dual importance of IP6 in supporting plant health and stress resilience, but also in fortifying crops against a range of pathogens [75]. They demonstrate that it is feasible to increase the iron content in crops without diminishing IP6 levels, thereby preserving its protective roles while also enhancing nutritional value [75]. The authors provide insights into how this approach applies to iron-deficiencies and related consequences, outlining a sustainable solution to nutritional deficiencies without compromising the intrinsic benefits of phytate. Stemming from the early anti-nutrient concerns of phytic acid intake, numerous efforts have been made to optimize strategies for reducing its content in food sources, with modern approaches involving the use of CRISPR-Cas9 genome editing to diminish phytic acid biosynthesis in plants [73]. However, others have cautioned that such reductions weaken the therapeutic potential of these foods [76], thus underscoring the need to balance mineral bioavailability with the preservation of phytic acid’s health-promoting properties.

While the movement toward lowering phytate content in food is still underway, a more nuanced framework is needed. Current literature largely reflects a general consensus that amid the anti-nutrient concerns, there are overt advantages to IP6 application in the realm of human health and disease. Others recognize the inherent balancing act rooted in IP6-related research: its mineral-chelating activity, once viewed as purely detrimental, also underlies a number of its health-promoting effects. This dual nature positions IP6 both as a dietary challenge to some, but to others a potent therapeutic asset, overall prompting scholars to call for deeper inquiry into its mechanisms and contextual uses in clinical settings. These modern publications and thorough reviews, including that of Pragya et al. (2025) [73], emphasize that future research must not only clarify the risk–benefit profile of phytic acid in diverse human populations, but also refine analytical techniques for its detection, reduction, and targeted application.

## 7. Concluding Remarks

These cumulative insights reflect a pivotal moment in the scientific understanding of IP6, as the long-standing antinutrient claims that historically dominated its narrative have been robustly contested. Results from in vitro experiments, animal models, and human trials have consistently unveiled IP6’s adaptive role in health promotion—ranging from its selective targeting of cancer cells to its potent regulatory role in inflammation, oxidative stress, and immune response. Clinical trials, though still sparse, have yielded encouraging results regarding IP6’s synergistic enhancement of chemotherapy efficacy and improvement of patients’ quality of life. Epidemiological findings strengthen this case, linking phytate-rich diets to the prevention of multiple pathogenic conditions.

This growing catalog of evidence compels the scientific and nutritional communities to look beyond the antiquated antinutrient chronicle and appreciate the path of discovery IP6 has paved. Decades of research have reshaped IP6’s legacy—from a misunderstood nutrient to a beacon of innovation in disease prevention and therapeutic promise. Properly assessing IP6 through the lens of modern evidence is essential, as it not only deepens our grasp on this multifaceted molecule, but also promotes a quest for innovative therapies that could transform patient outcomes and offer new hope for healing. One must weigh between risk versus benefit—what are the real risks of IP6 to human health, if any, versus the benefits? Do the presumed risks outweigh the benefits? Through further exploration into its mechanisms and applications across a spectrum of health contexts, investigators and clinicians can further validate IP6’s value in disease prevention and treatment.

## Data Availability

No new data were created or analyzed in this study.
